# Metabolic reprogramming regulated by TRAF6 contributes to the leukemia progression

**DOI:** 10.1038/s41375-024-02245-3

**Published:** 2024-04-12

**Authors:** Shinichiro Matsui, Chihiro Ri, Lyndsey C. Bolanos, Kwangmin Choi, Asuka Shibamiya, Arata Ishii, Koji Takaishi, Nagisa Oshima-Hasegawa, Shokichi Tsukamoto, Yusuke Takeda, Naoya Mimura, Akihide Yoshimi, Koutaro Yokote, Daniel T. Starczynowski, Emiko Sakaida, Tomoya Muto

**Affiliations:** 1 Department of Hematology, Chiba University Hospital, Chiba, Japan; 2 Department of Endocrinology, Hematology and Gerontology, Chiba University Graduate School of Medicine, Chiba, Japan; 3 Division of Experimental Hematology and Cancer Biology, Cincinnati Children’s Hospital Medical Center, Cincinnati, OH USA; 4 Department of Transfusion Medicine and Cell Therapy, Chiba University Hospital, Chiba, Japan; 5 Division of Cancer RNA Research, National Cancer Center Research Institute, Tokyo, Japan; 6 Department of Cancer Biology, University of Cincinnati, Cincinnati, OH USA; 7 Department of Pediatrics, Cincinnati Children’s Hospital Medical Center, Cincinnati, OH USA

**Keywords:** Acute myeloid leukaemia, Cancer metabolism

## Abstract

TNF receptor associated factor 6 (TRAF6) is an E3 ubiquitin ligase that has been implicated in myeloid malignancies. Although altered TRAF6 expression is observed in human acute myeloid leukemia (AML), its role in the AML pathogenesis remains elusive. In this study, we showed that the loss of TRAF6 in AML cells significantly impairs leukemic function in vitro and in vivo, indicating its functional importance in AML subsets. Loss of TRAF6 induces metabolic alterations, such as changes in glycolysis, TCA cycle, and nucleic acid metabolism as well as impaired mitochondrial membrane potential and respiratory capacity. In leukemic cells, TRAF6 expression shows a positive correlation with the expression of O-linked N-acetylglucosamine (O-GlcNAc) transferase (OGT), which catalyzes the addition of O-GlcNAc to target proteins involved in metabolic regulation. The restoration of growth capacity and metabolic activity in leukemic cells with TRAF6 loss, achieved through either forced expression of OGT or pharmacological inhibition of O-GlcNAcase (OGA) that removes O-GlcNAc, indicates the significant role of O-GlcNAc modification in the TRAF6-related cellular and metabolic dynamics. Our findings highlight the oncogenic function of TRAF6 in leukemia and illuminate the novel TRAF6/OGT/O-GlcNAc axis as a potential regulator of metabolic reprogramming in leukemogenesis.

## Introduction

Acute myeloid leukemia (AML) is a clonal hematopoietic stem and progenitor cell (HSPC) malignancy defined by the overproduction of immature myeloid cells (blasts) associated with a differentiation block. Although 35–40% of patients with AML aged ≤60 years achieve 5-year survival because of the progress in understanding the biology and treatment of AML, a considerable number of patients with AML still suffer from refractory/relapsed AML [1, 2]. In addition, the outcome for the AML aged >60 years remains poor, which is 5-15% of 5-year survival rate [1, 2]. AML development is propelled by genetic and epigenetic changes affecting crucial genes, including those for DNA methylation and signaling pathways [3–6]. Notably, the presence of some driver mutations associated with AML in healthy, particularly older, individuals is indicative of a pre-leukemic state known as clonal hematopoiesis of indeterminate potential (CHIP), which increases the risk of developing myeloid malignancies [7–10].

Tumor necrosis factor (TNF) receptor-associated factor 6 (TRAF6), an E3 ubiquitin ligase, plays a pivotal role in mediating innate immune signaling, primarily through Toll-like receptor (TLR) pathways. Upon TLR activation, TRAF6 undergoes K63-linked autopolyubiquitination, leading to the activation of various signaling cascades, including the NF-κB pathway [11]. Futhremore, TRAF6 interacts with a variety of proteins and has several substrates, such as AKT1 [12], p62 [13] and SMURF1 [14], which are outside the innate immune signaling pathway. While TRAF6 is critical for immune cell activation, its functions extend beyond immunoregulation to include roles in cellular growth and survival, bone homeostasis, lymph node organogenesis, and mammary gland development [11].

Recent research has uncovered TRAF6’s complex role in myeloid malignancies, illustrating its dual capacity as both an oncogene and a tumor suppressor [15–17]. Increased TRAF6 signaling is linked to myelodysplastic syndrome (MDS), where TRAF6 overexpression in mouse HSPCs results in MDS-like features, such as cytopenia, by regulating exon specification through TRAF6-mediated ubiquitination of RNA-binding protein hnRNPA1 [18]. Furthermore, activation of TLR-TRAF6 signaling by chronic inflammation in MDS increases the competitive advantage of HSPCs harboring MDS mutations. This is achieved through the upregulation of the ubiquitin-modifying enzyme A20 and a switch from canonical to non-canonical NF-κB signaling, indicating that TRAF6 functions as an oncogene in MDS [19].

Conversely, TRAF6’s tumor suppressive function is evident in different models of myeloid neoplasms. Specifically, overexpression of TRAF6 in a FLT3-ITD mouse model inhibited MPN development [20]. On the other hand, its absence in mice deficient for the *Tet2* gene— a representative target gene associated with CHIP mutations in humans—led to leukemic transformation from pre-leukemic cells. This transformation was notably due to reduced ubiquitination and the consequent lack of functional repression of MYC, as TRAF6 directly ubiquitinates and negatively regulates MYC activity [20]. This underscores TRAF6’s tumor suppressive role and highlights the importance of a nuanced understanding of TRAF6’s diverse impacts. It is essential to consider the specific type of myeloid neoplasm and its context, including mutations and whether the disease is in the pre-leukemic or leukemic stage. Notably, the role of TRAF6 in hematopoietic cells during the leukemic phase has not been evaluated so far, leaving a critical gap in our understanding of its function in AML progression.

Therefore, this study aims to delineate TRAF6’s role during the leukemic phase by investigating the effects of TRAF6 loss in leukemic cells, both in vitro and in vivo. We propose that TRAF6 acts as a regulator of metabolic reprogramming, a crucial mechanism in AML progression, offering novel insights into its multifaceted role in leukemia advancement.

## Materials and methods

### Mice

Traf6^fl/fl^ mice (C57Bl/6) were a kind gift from Dr. Yongwon Choi (University of Pennsylvania) [21]. Traf6^fl/fl^ mice were crossed with Mx1-Cre mice (Jackson Laboratory, 003556) and R26-CreERT2 mice (Jackson Laboratory, 008463) for inducible deletion. All mice were bred, housed and handled in the Association for Assessment and Accreditation of Laboratory Animal Care-accredited animal facility of Cincinnati Children’s Hospital Medical Center (CCHMC). Experimental protocols were approved by the CCHMC Institutional Animal Care and Use Committee.

### Cell lines

HEL, THP-1, TF-1, MOLM14 and MV4;11 cells were purchased from American Type Culture Collection (ATCC). The cells were cultured in RPMI1640 medium with 10% fetal bovine serum (FBS) and 1% penicillin and streptomycin (P/S). 10 ng/mL of human IL-3 (578006. Biolegend) was added to the medium for culturing TF-1. HEK 293 T cells was purchased from ATCC, and were cultured in DMEM with 10% FBS and 1% P/S. All these cell lines were cultured at 37 °C with 5% CO_2_. All the cell lines were authenticated using short tandem repeat profiling and tested for mycoplasma contamination once every 6 months.

### Generation of MLL-AF9 AML mouse model

To generate a *Traf6*-deficient *MLL-AF9* model, *Traf6*^*+/+*^*;Mx1-Cre*, *Traf6*^*fl/fl*^*;Mx1-Cre*, *Traf6*^*+/+*^*;R26-CreERT2* and *Traf6*^*fl/fl*^*;R26-CreERT2* lineage negative (Lin^−^) BM cells from C57Bl/6 mice were transduced with retrovirus encoding MLL-AF9 and a GFP reporter [22]. Due to the low transduction efficiency of these cells, three rounds of colony replating were performed to enrich for MLL-AF9-expressing AML cells. By the third plating, we confirmed that nearly all colonies express MLL-AF9 as determined by FACS ( > 95% GFP^+^ cells). Five hundred thousand *Traf6*^*+/+*^*;Mx1-Cre* or *Traf6*^*fl/fl*^*;Mx1-Cre* MLL-AF9-expressing (GFP^+^) AML cells were transplanted into lethally irradiated recipient mice (CD45.1^+^B6.SJL^Ptprca Pep3b/Boy^; 6-10 weeks of age) together with 500,000 CD45.1^+^B6.SJL^Ptprca Pep3b/Boy^ helper BM cells. At 2 weeks after transplantation, the recipient mice were injected with 200 mL of polyinosinic-polycytidylic acid (pIpC) (Tocris Bioscience, Cat#4287) dissolved in phosphate-buffered saline (PBS) at a concentration of 1.5 mg/mL intraperitoneally every other day 5 times to delete *Traf6*. For in vitro analysis, such as evaluation for proliferative capacity, gene expression profile, cell cycle, mitochondrial membrane potential, intracellular glucose level, glucose uptake capacity, and extracellular flux analysis, *Traf6* was deleted in vitro. *MLL-AF9;Traf6*^*+/+*^*;R26-CreERT2* and *MLL-AF9;Traf6*^*fl/fl*^*;R26-CreERT2* leukemic cells were cultured with IMDM (Wako, Cat#098-0645) with 10% FCS, 10 ng/mL of SCF (Peprotech, Cat#250-03), 10 ng/mL of IL-3 (Peprotech, Cat#213-13), 10 ng/mL of IL-6 (Peprotech, 200-06), and 1 μM 4-hydroxytamoxifen (4-OHT) (Sigma, Cat# H7904) for 4 days. Efficient deletion of *Traf6* was confirmed by immunoblotting and RNA sequencing.

### Extracellular flux analysis

Leukemic cells were seeded into a 96-well plate precoated with Cell-Tak (354240, Lot 2122006, Corning). The plate was centrifuged for 1 min at 300 × g and analyzed with the Agilent Seahorse XF Cell Mito Stress Test Kit (103015-100, Agilent technologies). During the assay, 1.5 mM Oligomycin, 1.5 mM FCCP, and 0.5 mM Rotenone/Antimycin A were added sequentially. Oxygen consumption rates and extracellular acidification rate (ECAR) were measured on Seahorse XFe96 Analyzer (Agilent Technologies) and data were analyzed using Wave software (Agilent Technologies).

### Measurement of mitochondrial membrane potential

Leukemic cells were incubated with tetramethylrhodamine ethyl ester (TMRE, ab113852, Abcam) at 37 °C for 20 min in concentration of 400 nM. Measurement of mitochondrial membrane potential was performed using FACSCanto II Flow Cytometer (BD biosciences), and data were analyzed by FlowJo software (BD biosciences).

### Metabolome analysis

The cell suspension was transferred to a tube and centrifuged to pellet the cells. Culture medium was aspirated from the tube, and the cells were washed with 10 ml of 5% mannitol solution. The cells were then treated with 800 µL of methanol and vortexed for 30 sec to suppress enzyme activity. Next, 550 µL of Milli-Q water containing internal standards (H3304-1002, Human Metabolome Technologies, Inc. (HMT), Tsuruoka, Yamagata, Japan) was added to the cell extract, which was vortexed for another 30 s. The extract was then centrifuged at 2300 × *g*, 4 °C for 5 min, after which 700 µL of the supernatant was centrifugally filtered through a Millipore 5-kDa cutoff filter (UltrafreeMC-PLHCC, HMT) at 9100 × *g*, 4 °C for 120 min to remove macromolecules. Subsequently, the filtrate was evaporated to dryness under vacuum and reconstituted in 50 µL of Milli-Q water for metabolome analysis at HMT. Metabolome analysis was conducted according to HMT’s ω Scan package, using capillary electrophoresis Fourier transform mass spectrometry (CE-FTMS) based on the methods described previously [23].

### Statistical analysis

The sample size for animal experiments was calculated using power analysis to ensure over 80% power at a 5% error rate. Comparisons between two groups were conducted using Student’s *t* test (unpaired, two-tailed) for normally distributed data. For data not following normal distribution, Welch’s *t* test was employed. The F-test was used to assess the normality of distribution. Results are expressed as mean ± standard deviation (SD). Neither randomization nor blinding was implemented in the study. All samples and animals were included in the analysis. Kaplan-Meier survival analysis was performed using the Mantel-Cox test. All graphs and statistical analyses were executed using GraphPad Prism software (version 10.1.2).

## Results

### Loss of TRAF6 impairs leukemic cell function in vitro and in vivo

**Fig. 1:**
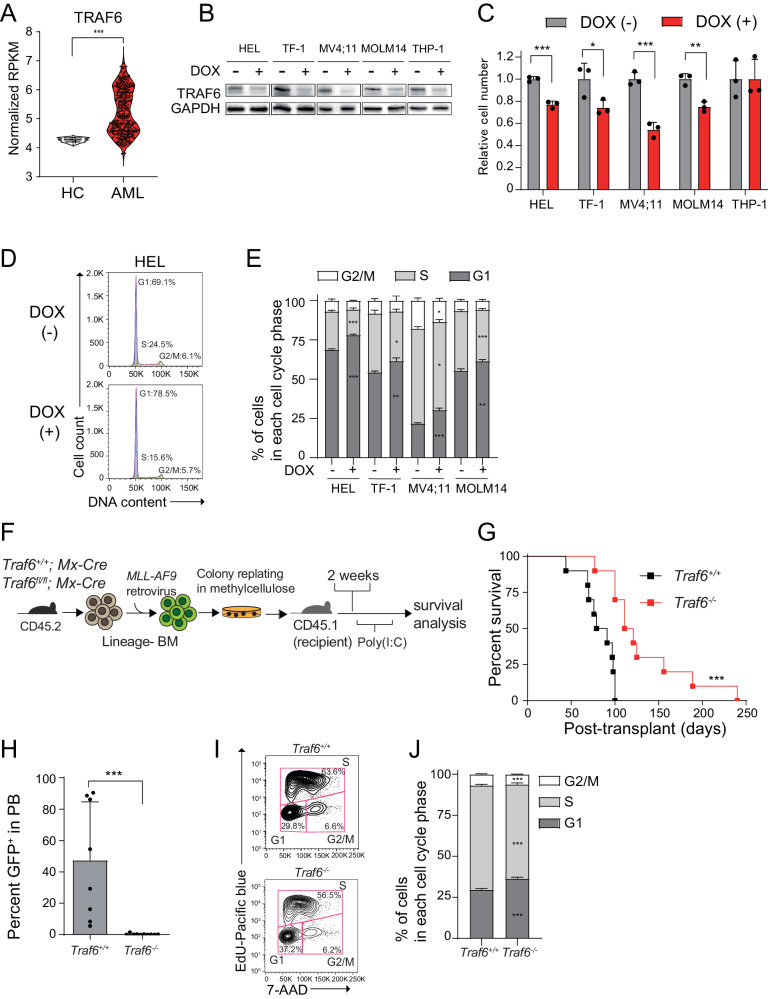
Oncogenic function of TRAF6 in AML. **A** TRAF6 mRNA expression in healthy BM CD34^+^ cells (n = 12) and AML (n = 451). The data for both healthy BM CD34+ cells and AML patients were retrieved from a published database (BeatAML) [24]. **B** Immunoblot analysis confirming knockdown of TRAF6 in leukemic cells upon addition of DOX(1 μg/mL). **C** Viable cell growth of HEL, TF-1, MV4;11, MOLM14 and THP-1 cells transduced with the inducible shTRAF6 was assayed by trypan blue exclusion. The relative cell number was evaluated on 7 days after equal number of the cells were seeded. Data are presented as the means ± SD from biological triplicates. Results are representative of three independent assays. **D** Representative flow cytometric analysis for the evaluation of cell cycle of HEL cells transduced with inducible shTRAF6. **E** Percentage of cells in each cell cycle phase, shown as means ± SD from biological replicates (n = 3). These results are representative of two independent assays. **F** Overview of experimental design to examine the requirement of TRAF6 for the *MLL-AF9* leukemic function in vivo. Isolated Lin^-^ BM cells were transduced with retrovirus encoding *MLL-AF9* and GFP. The transduced BM cells were serially replated in methylcellulose medium to select transformed cells. Lethally-irradiated recipient mice (CD45.2) were engrafted with the transformed BM cells along with wild-type BM cells (CD45.1) for radioprotection. From day 14, the recipient mice received intraperitoneal injection of polyinosinic-polycytidylic acid [poly(I:C)] to delete TRAF6, and then were monitored for engraftment and overall survival. **G** Kaplan-Meier analysis of overall survival of mice engrafted with *MLL-AF9*;*Traf6*^*+/+*^ (n = 10) and *MLL-AF9;Traf6*^*−/−*^ (n = 10) AML cells. **H** Summary of the leukemic cell burden (GFP^+^) in the PB of the mice 10 weeks after transplant with *MLL-AF9;Traf6*^*+/+*^ (n = 8) and *MLL-AF9;Traf6*^*−/−*^ (n = 10) AML cells. **I** Representative flowcytometric profiles from EdU assay using *MLL-AF9;Traf6*^*+/+*^ and *MLL-AF9;Traf6*^*−/−*^ AML cells. **J** Percentage of cells in each cell cycle phase, shown as means ± SD for biological replicates (n = 6). HC, healthy control. *P < 0.05; **P < 0.01, ***P < 0.001.

To examine the role of TRAF6 in AML cells, we first compared the expression of *TRAF6* in bone marrow (BM) cells from AML patient samples with that of healthy controls using a publicly available database [24]. RNA-seq analysis revealed that the mRNA levels of TRAF6 in AML were higher compared with that in healthy controls (Fig. 1A), suggesting the potential importance of TRAF6 in leukemogenesis. We previously demonstrated that the loss of TRAF6 in BM cells with a deficiency of Tet2, which is the most frequently observed CHIP-associated mutation, results in transformation to leukemia in mice [20]. However, the role of TRAF6 in leukemia cells, rather than transformation from CHIP-associated mutants, remains unclear. We addressed this by knocking down TRAF6 in various human AML cell lines, including HEL, TF-1, MV4;11, MOLM14, and THP-1, using a doxycycline (DOX)-inducible shRNA system. TRAF6 knockdown notably inhibited proliferation in all tested cell lines except THP-1 (Fig. 1B, C). To identify the basis for the reduced cell number in TRAF6-knockdown leukemia cells, we determined the effect of TRAF6 loss on apoptosis and cell cycle dynamics. Cell cycle analysis revealed that the loss of TRAF6 led to an increased proportion of cells in the G1 phase and a decreased proportion in the S phase among HEL, TF-1, MV4;11, and MOLM14 cells (Fig. 1D-E). Conversely, TRAF6 loss did not significantly impact apoptosis in these cells (data not shown). These observations suggest that the inhibitory effects of TRAF6 loss on leukemic cell proliferation stem from altered cell cycle progression rather than from increased apoptosis. To validate these findings in vivo, we established a myeloid leukemia model induced by the leukemic fusion gene *MLL-AF9* [22]. Isolated lineage^−^ (Lin^−^) BM cells, derived from *TRAF6*^*+/+*^*;MxCre* and *TRAF6*^*flox/flox*^*;MxCre* mice [25], were retrovirally transduced with vectors expressing *MLL-AF9* (MSCV-IRES-GFP) and then serially replated in methylcellulose medium to select transformed cells. The transformed BM cells (CD45.2^+^) were transplanted into lethally-irradiated recipient mice along with wild-type BM cells (CD45.1^+^). At day 14 post-transplant, TRAF6 was deleted by intraperitoneally injecting polyinosinic-polycytidylic acid (pIpC) (Fig. 1F). As expected, the mice engrafted with *MLL-AF9;Traf6*^*+/+*^ cells developed a rapid and fully penetrant AML up to 100 days post-transplantation (Fig. 1G). In contrast, mice engrafted with *MLL-AF9;Traf6*^*−/−*^ cells developed a significantly delayed leukemia (Fig. 1G). Consistent with these findings, an immunophenotypic analysis of peripheral blood revealed a reduced leukemic burden in the mice engrafted with *MLL-AF9; Traf6*^*−/−*^ cells compared with those engrafted with *MLL-AF9;Traf6*^*+/+*^ cells at the time point (Fig. 1H). Cell cycle assessments in murine *MLL-AF9* leukemic cells confirmed that TRAF6 loss impairs cell cycle progression as observed in human leukemic cells (Fig. 1I–J). Together, these findings affirm that TRAF6 loss suppresses leukemic cell function both in vitro and in vivo.

### TRAF6 expression in AML is inversely correlated with mitochondria-related gene signatures

**Fig. 2:**
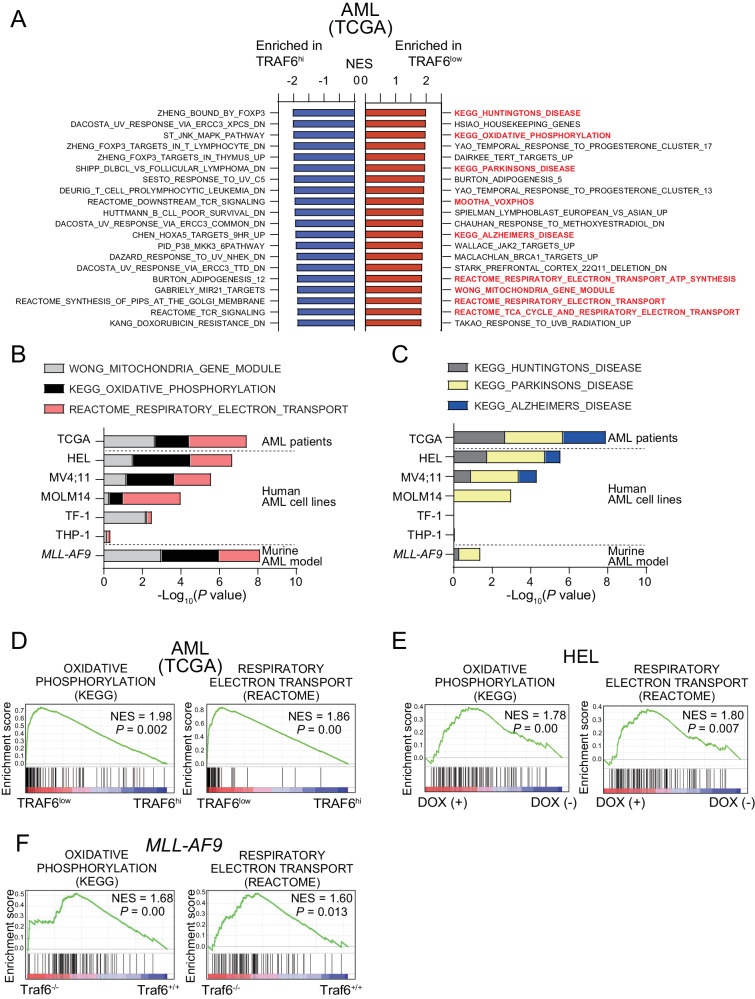
TRAF6 expression inversely correlates with mitochondrial function-related gene signatures in AML. **A** Normalized enrichment scores (NES) from Gene Set Enrichment Analysis (GSEA) for the top 20 upregulated (red, top) and downregulated (blue, bottom), significantly altered gene sets in TRAF6^low^ compared to TRAF6^hi^ AML patient samples from the TCGA AML dataset [3]. Low and high TRAF6 expressions defined by: TRAF6^low^, Z score <1; TRAF6^hi^, Z score >1. **B**, **C** RNA sequencing analysis of two groups: (1) human leukemic cell lines with inducible shTRAF6, treated with or without doxycycline (DOX, 1 μg/mL) for 7 days, and (2) *MLL-AF9;Traf6*^*+/+*^ and *MLL-AF9;Traf6*^*−/−*^ leukemic cells. The mitochondrial states were evaluated using GSEA profiles based on TRAF6^low^ leukemic cells, with a focus on mitochondria-associated gene signatures organized by their *P* values (log_10_). Selected gene set enrichment plots of AML patients stratified based on low/high TRAF6 expression (**D**), HEL cells transduced with the inducible shTRAF6 (**E**), and *MLL-AF9;Traf6*^*+/+*^ and *MLL-AF9;Traf6*^*−/−*^ leukemic cells (**F**). NES normalized enrichment score, DOX doxycyclin.

To determine the molecular mechanism of the inhibitory effects of TRAF6 loss on leukemia function, we stratified AML patient samples based on TRAF6 expression and compared the gene expression profiles between AML with high (Z score >1.0, hereafter TRAF6^hi^ AML) and low (Z score <1.0, hereafter TRAF6^low^ AML) TRAF6 expression. TRAF6 is not only a central mediator of innate immune signaling, but is also involved in other immune functions, such as T cell receptor (TCR) signaling and immune control mediated by regulatory T cells [26, 27]. As expected, gene set enrichment analysis revealed that immune-related gene sets, such as those associated with FOXP3 targets, MAPK signaling, and TCR signaling, were significantly enriched in TRAF6^hi^ AML cells (Fig. 2A). Interestingly, the most enriched gene sets in TRAF6^low^ AML cells included mitochondrial function-associated processes, such as oxidative phosphorylation (OXPHOS), respiratory electron transport, and the TCA (tricarboxylic acid) cycle (Fig. 2A, B, D). Furthermore, gene sets related to neurodegenerative diseases, such as Huntington’s, Parkinson’s, and Alzheimer’s disease, of which mitochondrial dysfunction plays a central role in pathogenesis, were also overrepresented in TRAF6^low^ AML cells (Fig. 2A, C) [28]. Similarly, knocking down TRAF6 in HEL, MV4;11, MOLM14, and TF-1 cells—but not in THP-1 cells—induced mitochondrial-related gene expression signatures (Fig. 2B, C, E). Notably, the absence of such enrichment in THP-1 cells aligns with our observations that TRAF6 loss did not impact their proliferation, suggesting a possible link between the mitochondrial gene expression profile and the proliferative capacity affected by TRAF6 status (Fig. 2B, C, E). This pattern was also present in murine *MLL-AF9;Traf6*^*−/−*^ leukemic cells (Fig. 2B, C, F). These results suggest that TRAF6 loss in AML may be linked to alterations in the expression of genes involved in mitochondrial processes.

### Loss of TRAF6 in AML results in the perturbation of mitochondrial function

**Fig. 3:**
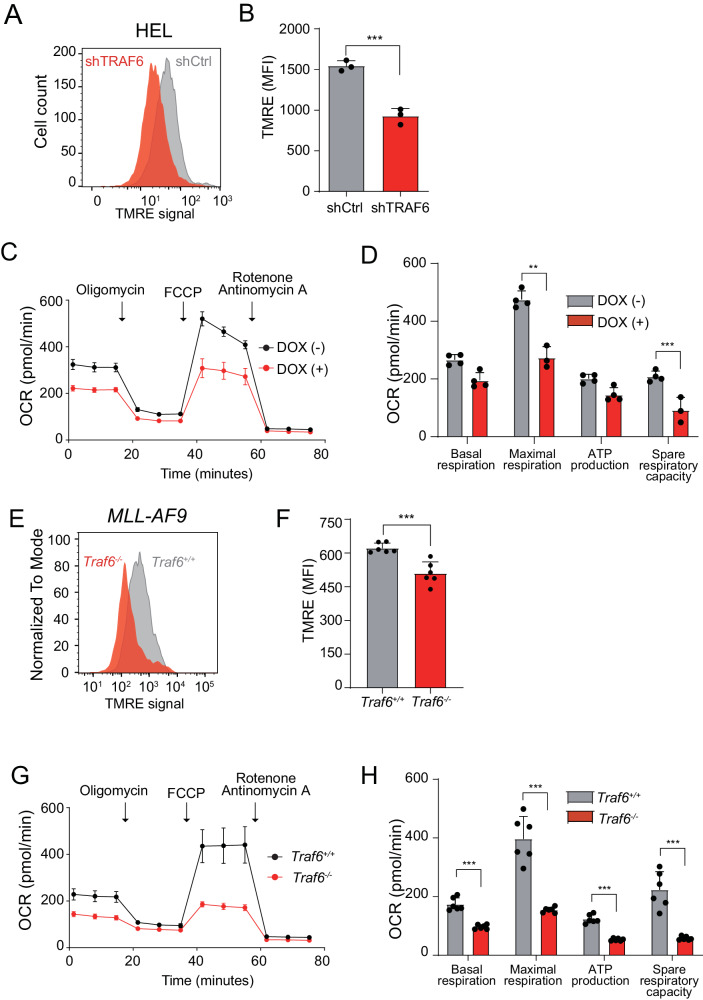
Loss of TRAF6 in leukemia cells induces the changes in the mitochondrial function parameters. **A** Representative flow cytometry histograms of mitochondrial TMRE levels in HEL cells expressing shTRAF6 or shControl (shCtrl). **B** Median fluorescent intensity (MFI) of tetramethylrhodamine ethyl ester (TMRE) observed from HEL cells expressing shTRAF6 or shCtrl. Data are presented as the means ± SD from biological replicates (n = 3). Results are representative of two independent assays. **C** Oxygen consumption rate (OCR) in HEL cells transduced with the inducible shTRAF6. Cells were sequentially treated with oligomycin, fluoro-carbonyl cyanide phenylhydrazone (FCCP), and rotenone/antimycin A at the indicted time points. Data are presented as the means ± SD from technical replicates (n = 4). Results are representative of three independent assays. **D** Basal respiration, maximal respiration, ATP production and spare respiratory capacities of HEL cells transduced with the inducible shTRAF6 calculated from the data of (**C**). Data are shown as the means ± SD (n = 4). **E** Representative flow cytometry histograms of mitochondrial TMRE levels in *MLL-AF9;Traf6*^*+/+*^ and *MLL-AF9;Traf6*^*−/−*^ leukemic cells. **F** MFI of TMRE observed from *MLL-AF9;Traf6*^*+/+*^ and *MLL-AF9;Traf6*^*−/−*^ leukemic cells. Data are presented as the means ± SD from biological replicates (n = 6). Results are representative of two independent assays. **G** OCR in *MLL-AF9;Traf6*^*+/+*^ and *MLL-AF9;Traf6*^*−/−*^ leukemic cells. Cells were sequentially treated with oligomycin, FCCP, and rotenone/antimycin A at the indicted time points. Data are presented as the means ± SD from technical replicates (n = 6). Results are representative of two independent assays. **H** Basal respiration, maximal respiration, ATP production and spare respiratory capacities of *MLL-AF9;Traf6*^*+/+*^ and *MLL-AF9;Traf6*^*−/−*^ leukemic cells calculated from the data of (G). Data are shown as the means ± SD (n = 6). **, *P* < 0.01; ***, *P* < 0.001.

Mitochondria are cellular organelles that generate energy and metabolites required for cell survival and growth. Energy in the form of adenosine triphosphate (ATP) is primarily generated in mitochondria by the OXPHOS process, in which byproducts of the TCA cycle feed the electron transport chain (ETC) complexes and their electrons pass through the ETC. As the electrons are funneled through the various complexes of the inner mitochondrial membrane, the ETC generates a mitochondrial membrane potential (MMP) that produces ATP [29]. To ascertain the impact of TRAF6 loss on mitochondrial function in AML, we assessed MMP using tetramethylrhodamine-ethyl ester dye in leukemia cells. Post TRAF6 knockdown, HEL and TF-1 cells exhibited a reduction in MMP compared to controls (Fig. 3A, B and Supplemental Fig. 1A). Further evaluation of mitochondrial function with an extracellular flux analyzer indicated a decrease in respiratory capacity in TRAF6-knockdown HEL and TF-1 cells (Fig. 3C, D and Supplemental Fig. 1B). Conversely, MV4;11 and MOLM14 cells did not exhibit notable changes in MMP or respiratory capacity (Supplemental Fig. 1C–F). This lack of change suggests that these cell lines might compensate for mitochondrial function disruption through the upregulation of mitochondrial genes, and that other molecular mechanisms may mitigate the effects of TRAF6 knockdown on their proliferation. Similarly, murine *MLL-AF9;Traf6*^*−/−*^ leukemic cells displayed a decreased MMP and reduced mitochondrial respiratory capacity compared to their *Traf6*^*+/+*^ counterparts (Fig. 3E–H). Collectively, these findings indicate that TRAF6 loss can lead to mitochondrial dysfunction in leukemic cells, contributing to the observed phenotypic alterations in a subset of AML cases.

### TRAF6 loss in AML gives rise to metabolic alterations

**Fig. 4:**
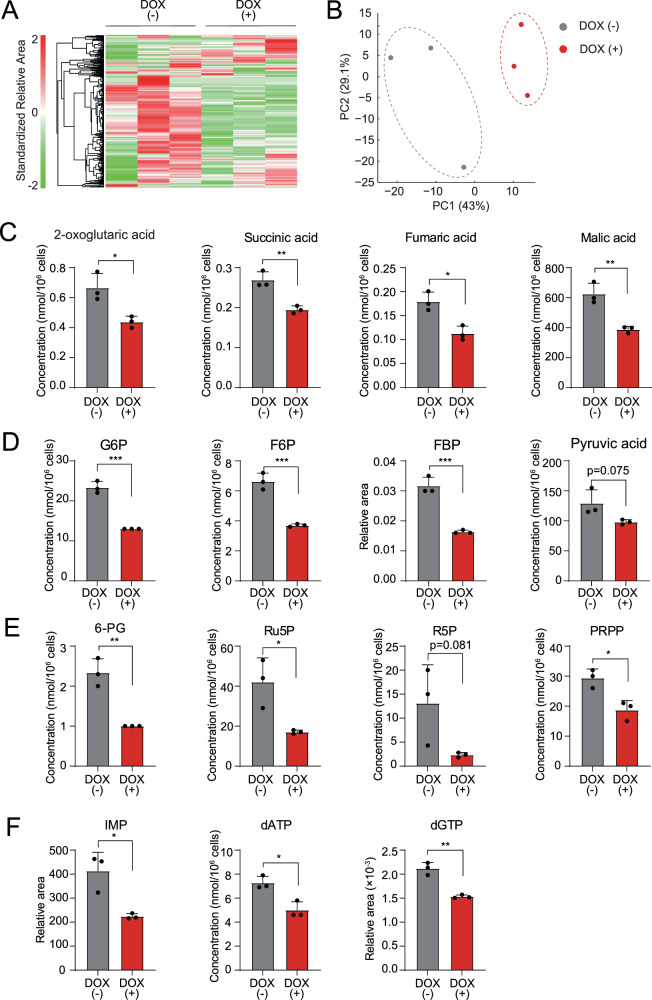
TRAF6 loss in leukemia leads to dynamic alteration of metabolic profile. **A–F** Metabolites in HEL cells transduced with the inducible shTRAF6 cultured for 2 days with or without DOX (1 µg/mL) were analyzed using capillary electrophoresis Fourier transform mass spectrometry (CE-FTMS) (n = 3). Hierarchical clustering heatmap analysis (**A**) and principal component analysis (**B**) of all metabolites that were detected as a peak by CE-FTMS (n = 483). The concentrations or relative area of selected metabolites in TCA cycle (**C**), glycolytic pathway (**D**), pentose phosphate pathway (**E**) and purine synthesis pathway (**F**). G6P glucose 6-phosphate, F6P fructose 6-phosphate, FBP fructose 1,6-diphosphate, 6-PG 6-phosphogluconic acid, Ru5P ribulose 5-phosphate, R5P ribose 5-phosphate, PRPP phosphoribosyl pyrophosphate, IMP inosine monophosphate, dATP deoxyadenosine triphosphate, dGTP deoxyguanosine triphosphate. *P < 0.05; **, <0.01; ***P < 0.001.

Mitochondria are essential organelles that act as metabolic hubs and signaling platforms within the cell. Therefore, we evaluated the effect of TRAF6 loss on the metabolic profiles of leukemia cells by metabolome analysis. Heatmap and principal component analysis revealed that TRAF6 loss in HEL cells induced dynamic changes in several metabolites (Fig. 4A, B). Since we observed reduced MMP and impaired mitochondrial respiratory capacity in TRAF6 knockdown leukemic cells (Fig. 3A–E), we first noted alterations in intermediates of the TCA cycle (Supplemental Fig. 2A). Consistent with our findings, the level of multiple metabolites in the TCA cycle, including 2-oxoglutaric acid, succinic acid, fumaric acid, and malic acid were reduced in TRAF6 knockdown HEL cells (Fig. 4C). For energy generation through the TCA cycle, glucose in the cytoplasm ultimately breaks down into pyruvic acid, which is transported to the mitochondria and metabolized into acetyl-CoA under aerobic conditions (Supplemental Fig. 2A). The level of most intermediates in the glycolytic pathway, including glucose 6-phosphate (G6P), fructose 6-phosphate, fructose 1,6-diphosphate, and pyruvic acid were reduced in TRAF6 knockdown HEL cells, suggesting a possible cause for the reduction of intermediates in the TCA cycle (Fig. 4D). Furthermore, TRAF6 loss resulted in the reduction of most intermediates in the pentose phosphate pathway (PPP), such as phosphogluconic acid, ribulose 5-phosphate, ribose 5-phosphate, and phosphoribosyl pyrophosphate (Fig. 4E). Because PPP plays important role in purine synthesis (Supplemental Fig. 2A), we examined the metabolites in the purine synthetic pathway. Indeed, the level of inosine monophosphate, deoxyadenosine triphosphate, and deoxyguanosine triphosphate, which acts as a precursor for nucleic acid synthesis during replication, was decreased upon TRAF6 loss (Fig. 4F). These reductions in a broad spectrum of metabolic intermediates led us to speculate that TRAF6 loss might suppress glucose uptake, thus affecting leukemic cell metabolism. However, assessments of glucose uptake and intracellular glucose levels in TRAF6-knockdown HEL and TF-1 cells revealed minimal changes in these parameters (Supplemental Fig. 3A, B). Furthermore, intracellular glucose level in murine *MLL-AF9;Traf6*^*−/−*^ leukemic cells was higher than in control cells (Supplemental Fig. 3A), suggesting that accumulation of unused intracellular glucose due to the impairment of cascade reactions in metabolic pathways. Glucose uptake capacity in murine *MLL-AF9;Traf6*^*−/−*^ leukemic cells was suppressed compared with controls (Supplemental Fig. 3B), implying a negative feedback effect for high intracellular glucose levels. These findings indicate that TRAF6 loss leads to a global reduction in metabolic intermediates through mechanisms independent of glucose uptake capacity. Typically, mitochondrial dysfunction prompts enhanced glycolysis to compensate for reduced ATP production by impaired OXPHOS [30]. However, the evaluation of extracellular acidification rate (ECAR) in TRAF6-knockdown HEL and TF-1 cells, as well as murine *MLL-AF9;Traf6*^*−/−*^ leukemic cells, revealed the absence of such compensatory action (Supplemental Fig. 3C). This suggests that the metabolic changes induced by TRAF6 loss in leukemic cells are mediated through mechanisms affecting a broad range of cellular processes. Since metabolic reprogramming is a hallmark of malignancy and crucial for supporting the heightened proliferation of tumor cells [31], our results imply that TRAF6 loss in leukemia cells induces metabolic alterations, contributing to their inhibited growth capacity.

### OGT is a potential mediator of metabolic reprogramming regulated by TRAF6 in leukemia

**Fig. 5:**
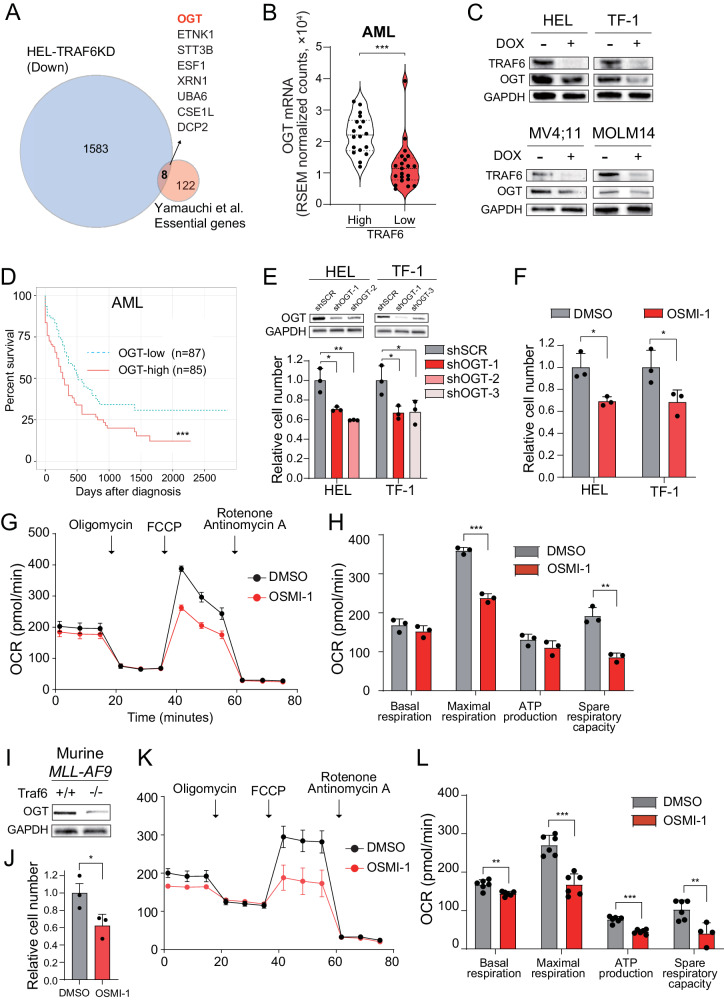
Similarity of the leukemic cellular features between loss of OGT and TRAF6. **A** Venn diagram of downregulated genes (1.5-fold, *P* < 0.05) in DOX-treated HEL cells transduced with the inducible shTRAF6 (relative to untreated HEL cells transduced with the inducible shTRAF6) and 130 AML essential genes identified by CRISPR-Cas9 screens [32]. **B** OGT mRNA expression in AML patients stratified on TRAF6 expression (TRAF6^hi^, n = 18; TRAF6^low^, n = 21) [3]. **C** Immunoblot analysis of TRAF6 and OGT in HEL, TF-1, MV4;11 and MOLM14 cells transduced with the inducible shTRAF6, treated with or without DOX (1 μg/mL) for 3 days. **D** Overall survival of AML patients stratified on OGT expression (OGT^hi^, n = 85; OGT^low^, n = 87) [3]. High and low OGT expressions defined by: OGT^hi^, above median; OGT^low^, below median. Survival curves were generated using the BloodSpot database (https://www.fobinf.com/). **E** Immunoblot analysis of OGT in HEL and TF-1 expressing shOGT or shSCR (Upper panel). Viable cell number of HEL and TF-1 expressing shOGT or shSCR was assayed by trypan blue exclusion. The relative cell number was evaluated 72 h after equal number of the cells were seeded. Data are presented as the means ± SD from technical triplicates. Results are representative of two independent assays. **F** Viable cell number of HEL and TF-1 exposed to 20 µM of OSMI-1 (OGT-inhibitor) was assayed by trypan blue exclusion. The relative cell number was evaluated 72 h after equal number of the cells were seeded. Data are presented as the means ± SD from technical triplicates. Results are representative of two independent assays. **G** OCR in HEL cells treated with OSMI-1 (20 μM) for 48 h. Cells were sequentially treated with oligomycin. FCCP, and rotenone/antimycin A at the indicted time points. Data are presented as the means ± SD from technical triplicates. Results are representative of two independent assays. **H** Basal respiration, maximal respiration, ATP production and spare respiratory capacities of HEL cells treated with OSMI-1 calculated from the data of (**G**). Data are presented as the means ± SD (n = 3). **I** Immunoblot analysis of OGT in *MLL-AF9;Traf6*^*+/+*^ and *MLL-AF9;Traf6*^*−/−*^ leukemic cells. **J** Viable cell number of *MLL-AF9* leukemic cells exposed to 2 µM of OSMI-1 was assayed by trypan blue exclusion. The relative cell number was evaluated 72 h after equal number of the cells were seeded. Data are presented as the means ± SD from technical triplicates. Results are representative of two independent assays. **K** *MLL-AF9* leukemic cells (2μM) for 48h. Cells were sequentially treated with oligomycin. FCCP, and rotenone/antimycin A at the indicted time points. Data are presented as the means ± SD from technical replicates (n = 6). Results are representative of two independent assays. **L **Basal respiration, maximal respiration, ATP production and spare respiratory capacities of *MLL-AF9* leukemic cells treated with OSMI-1 calculated from the data of (K). Data are presented as the means ± SD (n = 6).*P < 0.05; **<0.01; ***P < 0.001.

To identify the mechanism of metabolic alterations driven by TRAF6 loss in leukemia, we compared the upregulated genes in TRAF6-knockdown HEL cells (1.5-fold, P < 0.05) (Supplemental Table 1) with the 130 previously identified essential genes for AML cell survival in vitro and in vivo [32]. We found that 8 genes overlapped, and O-GlcNAc transferase (*OGT*) and *ETNK1* were associated with mitochondrial function and leukemia cell survival (Fig. 5A) [33, 34]. Stratification of AML patients revealed that *TRAF6* gene expression in human AML patient samples was positively correlated with the expression of *OGT*, but not *ETNK1* (Fig. 5B and data not shown). Correspondingly, TRAF6 knockdown in HEL and TF-1 cells also led to reduced OGT protein levels (Fig. 5C). Intriguingly, in MV4;11 and MOLM14 cells, where TRAF6 loss did not significantly affect mitochondrial function (Supplemental Fig. 1C–F), OGT expression was also suppressed (Fig. 5C), suggesting a broader positive correlation between TRAF6 and OGT expression in leukemic cells. Furthermore, stratification of AML patients revealed that OGT-low patients were associated with longer overall survival (*P* < 0.001) (Fig. 5D). This led us to investigate OGT as a mediator of TRAF6-regulated mitochondrial function in leukemia cells. To evaluate the role of OGT in leukemia progression, we examined the impact of OGT loss or inhibition on leukemic cell growth. Inducing OGT-targeted shRNAs in HEL and TF-1 cells significantly reduced cell numbers (Fig. 5E). Furthermore, treatment with the OGT inhibitor OSMI-1 suppressed cell growth in the leukemic cells, correlating with diminished mitochondrial respiratory function (Fig. 5F–H). Similar trends were observed in murine *MLL-AF9* leukemic cells, showing a positive correlation between Traf6 and Ogt levels, and susceptibility to OSMI-1, along with changes in mitochondrial respiratory function (Fig. 5I–L). These findings suggest that OGT loss mimics TRAF6 loss effects on leukemia cell metabolism and growth capacity.

### O-GlcNAc modification is a potential contributor to TRAF6-mediated metabolic reprogramming and leukemia progression

**Fig. 6:**
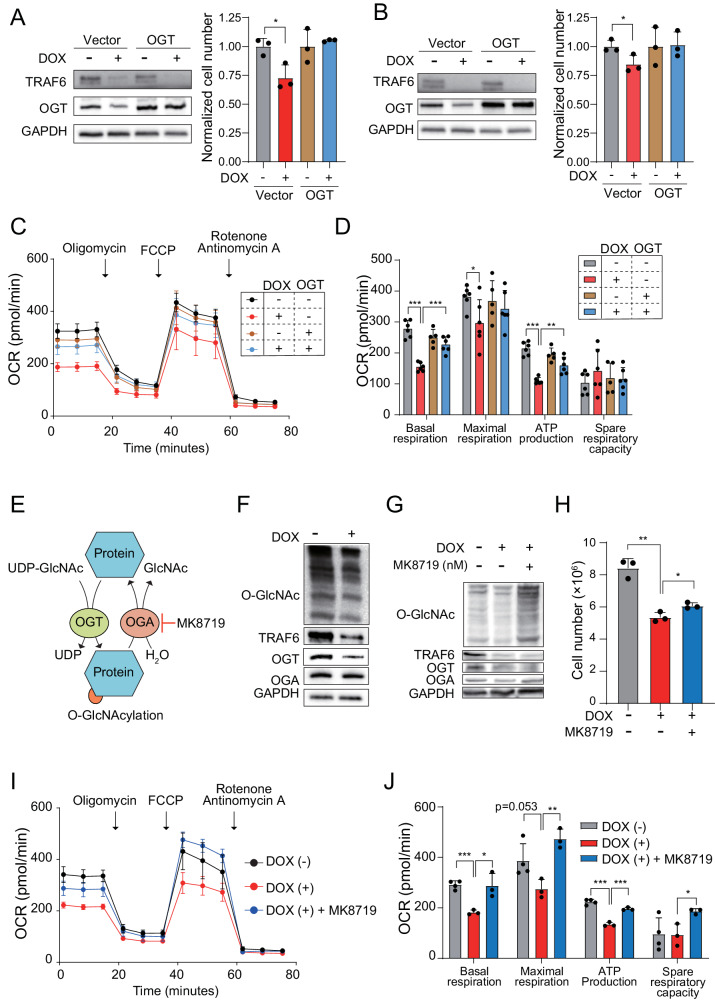
O-GlcNAc modification is a potential contributor for TRAF6-mediated metabolic reprogramming of leukemia. Immunoblot analysis of OGT in HEL (**A**) and TF-1 (**B**) transduced with inducible shTRAF6 expressing either control vector or cDNA of OGT, cultured with or without DOX (1 μg/mL) for 3 days (left panel). Viable cell growth of the cells was assayed by trypan blue exclusion (right panel). The normalized cell count, relative to untreated cells, was determined 72 h post-seeding of an equal number of cells. Data are presented as the means ± SD from technical triplicates. Results are representative of two independent assays. **C** OCR in HEL cells transduced with inducible shTRAF6, expressing control vector or cDNA of OGT, untreated or treated with DOX for 3 days. Cells were sequentially treated with oligomycin. FCCP, and rotenone/antimycin A at the indicted time points. Data are shown as the means ± SD for technical replicate analyses (n = 6). Results are representative of two independent assays. **D** Basal respiration, maximal respiration, ATP production and spare respiratory capacities of HEL cells calculated from the data of (**C**). The data are shown as the means ± SD (n = 6). **E** Schematic of O-GlcNacylation. **F** Immunoblotting of HEL cells transduced with the inducible shTRAF6, treated with or without DOX (1 μg/mL) for 3 days. **G** Immunoblotting of HEL cells transduced with the inducible shTRAF6, untreated with DOX, treated with DOX, and treated with DOX and 100 nM of MK8719 (OGA inhibitor). **H** One hundred thousand HEL cells transduced with inducible shTRAF6 were cultured with 1 μM of MK8719 for 7 days. Viable cell growth of the cells was assayed by trypan blue exclusion. Data are presented as the means ± SD for technical triplicates. Results are representative of two independent assays. **I** OCR in HEL cells transduced with the inducible shTRAF6 untreated with DOX, treated with DOX (1 μg/mL), and treated with DOX (1 μg/mL) and 200 nM of MK8719 (OGA inhibitor). Cells were sequentially treated with oligomycin. FCCP, and rotenone/antimycin A at the indicted time points. Data are presented as the means ± SD from technical replicate analyses (n = 3–4). Results are representative of two independent assays. **J** Basal respiration, maximal respiration, ATP production and spare respiratory capacities of HEL cells transduced with the inducible shTRAF6 calculated from the data of (**I**). Data are shown as the means ± SD (n = 3–4). *P < 0.05; **<0.01; ***P < 0.001.

To investigate OGT’s role in the regulation of mitochondrial function mediated by TRAF6 in leukemia, we utilized a lentiviral system to overexpress OGT in TRAF6 knockdown human leukemic cells, examining its impact on growth capacity. The forced overexpression of OGT successfully counteracted inhibitory effect of TRAF6 loss on the proliferation defect in HEL and TF-1, along with a corresponding recovery in mitochondrial respiratory capacity (Fig. 6A–D). This result suggests a link between OGT and the metabolic dysregulation due to TRAF6 loss, affecting leukemic cell proliferation. However, in murine *MLL-AF9;Traf6*^*−/−*^ leukemic cells, OGT overexpression did not rectify the proliferation defect (Supplemental Fig. 4A). This observation aligns with previous research indicating that both excessive and insufficient levels of OGT can negatively impact cell metabolism and proliferation [35].

OGT catalyzes the addition of an O-GlcNAc moiety to serine or threonine residues of protein substrates, while O-GlcNAcase (OGA) removes these modifications (Fig. 6E) [36]. Notably, the level of O-GlcNAc modification was reduced in TRAF6-knockdown HEL and TF-1 cells, as well as in murine *MLL-AF9;Traf6*^*−/−*^ leukemic cells (Fig. 6F and Supplemental Fig. 4B). Given that numerous metabolic enzymes are O-GlcNAc targets [36, 37], we hypothesized that the reduction in this modification disrupts metabolic reprogramming caused by TRAF6 loss in leukemia. To test this hypothesis, we examined the effects of the OGA inhibitor MK8719 on the growth capacity of leukemia cells impacted by TRAF6 loss. MK8719 treatment in TRAF6-knockdown HEL cells and murine *MLL-AF9;Traf6*^*–/*–^ leukemic cells restored O-GlcNAc levels (Fig. 6G and Supplemental Fig. 4C) and inhibited the effects of TRAF6 loss on growth capacity, correlating with a recovery in mitochondrial respiratory function (Fig. 6H–J and Supplemental Fig. 4D-F). However, MK8719 did not correct the proliferation defect in TRAF6-knockdown TF-1 cells (data not shown). Given that a broad range of metabolic enzymes are targets of O-GlcNAcylation, the metabolic status in leukemia cells is likely finely regulated by the balance between OGT and OGA expression and activity.

Taken together, these results suggest that the fine-tuned and complex regulation of O-GlcNAc modification plays an influential role in TRAF6-mediated metabolic reprogramming of leukemic cells.

## Discussion

The role of TRAF6 in myeloid malignancies is complex. Overexpression of TRAF6 occurs in HSPCs from MDS patients, and correspondingly, hematopoietic-specific TRAF6 overexpression in mice results in an MDS-like phenotype [18, 38]. Conversely, TRAF6 expression is diminished in MPN and a subset of AML patients. Furthermore, the absence of TRAF6 in *Tet2*-deficient pre-leukemic cells has been shown to promote the transition to overt leukemia in mice [20]. Our finding, demonstrating elevated TRAF6 expression in AML, led us to further explore TRAF6’s role in AML models, both in vitro and in vivo. These investigations have revealed that TRAF6 loss impedes AML progression, aligning with the oncogenic function of TRAF6 observed in our current study. Meanwhile, as described above, the previous study has suggested a tumor suppressive role in a *Tet2*-deficient pre-leukemic context. This contrast underscores the complexity of TRAF6’s function, which may vary significantly depending on the specific disease type and context. Such variability in function is not solely observed in TRAF6 but is also a characteristic of other cancer-associated genes, including *TP53* [39] and *EZH2* [40, 41]. Future research should focus on delineating TRAF6’s impact across a broad spectrum of diseases and their progression stages, including various contexts of CHIP, not limited to *TET2*-deficiency. This multifaceted approach is crucial to fully grasp TRAF6’s contributions to the dynamics of myeloid malignancies and to elucidate its potential context-dependent roles across different phases of disease development.

TRAF6 is an E3 ligase involved in innate immune signaling pathway activation. We previously demonstrated that TRAF6 expression in MDS cells is associated with the upregulation of inflammatory and immune-related genes [19]. TLR-TRAF6 signaling is activated in MDS cells and exhibits an altered response to chronic inflammation. This response contributes to a competitive advantage for MDS cells over normal HSPCs through non-canonical NF-κB signaling activation [19]. In the present study, TRAF6 expression in AML minimally affected inflammatory and immune-related gene expression (Fig. 2A), suggesting that TRAF6 expression has a differential impact on the gene expression profiles between AML and MDS. Therefore, a molecular mechanism mediated by TRAF6 other than the innate immune pathway activation may be involved in the pathogenesis of AML. Our in vitro validation experiments identified a novel role of TRAF6 in the regulation of metabolic reprogramming in AML cells. Gene expression analysis of publicly available datasets representing human AML samples revealed that reduced expression of TRAF6 is associated with mitochondrial function-related and disorder-related gene sets, such as Huntington’s, Parkinson’s, and Alzheimer’s diseases (Fig. 2A). Induction of shRNA targeting TRAF6 in leukemia cells resulted in the impairment of mitochondrial function as evidenced by a reduction of MMP, basal respiration, maximal respiration, and ATP production (Fig. 3 and Supplemental Fig. 1). Consistent with these findings, metabolome analysis revealed that the levels of most metabolites in the TCA cycle were decreased in TRAF6 knockdown leukemic cells (Fig. 4C). Interestingly, most metabolites in the glycolytic pathway, which converts glucose to pyruvate and acetyl-CoA as the first step in the TCA cycle in the mitochondria, were also reduced (Fig. 4D). Furthermore, metabolome analysis revealed that the nucleotide biosynthesis pathway, which is downstream of the PPP, is impaired during TRAF6 loss (Fig. 4F). Altogether, these observations suggest that TRAF6 loss in AML results in dynamic changes in metabolites and results in the disruption of metabolic reprogramming in leukemic cells.

The mechanism through which alterations in the metabolites of leukemic cells during TRAF6 loss impair cell growth is an important issue. Metabolic reprogramming is a hallmark of cancer cells to support the increased energy required for continuous growth and rapid proliferation [42]. For example, AML cells have high glycolytic activity and increased levels of anabolic precursors, such as intermediates of glycolysis, the PPP, and the TCA cycle, which also occurs in leukemia cells [43]. These metabolites are required for the production of energy, nucleotides, amino acids, and fatty acids, which contribute to the rapid proliferation of leukemia cells [43]. Indeed, cell cycle analysis in this study indicated that TRAF6 loss in leukemic cells resulted in a reduction of cell cycle progression (Fig. 1D, E, I–J). Therefore, our results suggest that the TRAF6 loss-mediated reduction of intermediates in various metabolic pathways results in a lack of fuel for the growth of leukemic cells.

Another possible mechanism resulting from TRAF6 loss on growth capacity involves chromatin modification and DNA methylation mediated by metabolites of the TCA cycle. For example, 2-oxoglutarate is an obligate co-substrate of Fe(II)/2-oxoglutarate-dependent dioxygenases (OGDD), which includes epigenetic modification enzymes, such as the ten-eleven translocation DNA demethylase and the Jumonji C domain-containing KDM2-7 histone demethylase. Thus, 2-oxoglutarate has a direct impact on gene expression by regulating histones and DNA demethylases [29]. Furthermore, succinate is considered an oncometabolite as evidenced by its accumulation in several cancers with inactivating mutations in succinate dehydrogenase [29]. Succinate is the product of OGDD-catalyzed reactions and when it accumulates, it acts as an antagonist of the reaction. Consequently, changes in succinate levels have profound effects on histones and DNA methylation, which subsequently results in gene expression changes [29]. Fumarate, another metabolite in the TCA cycle, is also a competitive inhibitor of multiple 2-oxoglutarate-dependent dioxygenases, including histone demethylases and the TET family [44–47]. Therefore, TRAF6 loss may give rise to epigenetic changes resulting from reduced levels of TCA cycle intermediates, which ultimately leads to an inhibitory effect on the proliferation of leukemic cells.

O-GlcNAc modification, catalyzed by OGT (introducer) and OGA (remover), is a significant posttranslational modification with a noted increase in cancer contexts [48, 49]. Previous studies identified OGT as an essential gene for AML survival [32] and our data show a positive correlation between OGT and TRAF6 transcript levels in AML. Given OGT’s role in maintaining hematopoietic stem cells by ensuring mitochondrial quality [34], we proposed that OGT loss-induced changes in O-GlcNAc modification could underlie the metabolic alterations associated with TRAF6 loss in AML. Forced expression of OGT or inhibition of OGA in TRAF6-knockdown AML cells restored mitochondrial respiratory capacity and countered growth suppression (Fig. 6), underscoring O-GlcNAc modification’s impact on cell metabolism. Numerous enzymes in pathways like glycolysis, the pentose phosphate pathway (PPP), and the TCA cycle are O-GlcNAc targets [36, 37]. Although the exact effects of O-GlcNAc modification on these enzymes in leukemic cells remain to be fully elucidated, existing studies indicate that O-GlcNAc modification of metabolic enzymes, such as G6P dehydrogenase and fumarate hydratase, can promote the growth of certain solid tumors by inducing metabolic and epigenetic changes [50, 51]. These insights suggest that OGT-induced O-GlcNAc modifications could be a potential molecular contributor to TRAF6-mediated metabolic reprogramming in leukemic cells.

A major gap in our understanding of TRAF6-mediated metabolic reprogramming in AML is a lack of understanding of TRAF6-mediated regulation of OGT expression. Although TRAF6 plays an important role in the innate immune pathway activation by K63-ubiquitination of TRAF6 itself, it also affects the function of other target proteins through K63-ubiquitination. For example, TRAF6-mediated K63-ubiquitination of FOXP3, which is a key transcription factor in the development and function of regulatory T cells (Tregs), promotes the nuclear accumulation of FOXP3 and contributes to Treg gene expression and function [26]. Human DNA2 nuclease/helicase is also K63-ubiquitinated by TRAF6 and ubiquitination promotes its nuclear localization [52]. In hepatocellular carcinoma cells, TRAF6 has oncogenic potential by interacting with and ubiquitinating HDAC3 with K63-linked ubiquitin chains, which leads to the dissociation of HDAC3 from the c-Myc promoter. This epigenetically enhances the expression of c-Myc mRNA [53]. It will be worth exploring whether these mechanisms, including those involving in epigenetic and transcription, affect the transcriptional regulation of OGT by TRAF6 in leukemic cells.

In summary, our study underscores the role of TRAF6 in modulating metabolic processes and cellular dynamics in leukemia. We suggest that O-GlcNAc modification, mediated by OGT, represents a significant molecular pathway in TRAF6-driven metabolic reprogramming. Identifying specific OGT target proteins in AML and further analyzing how such modifications influence protein function are essential next steps. These efforts will enhance our understanding of the TRAF6-OGT-O-GlcNAc axis in leukemia, potentially opening new avenues for therapeutic intervention.

### Supplementary information

Supplemental method

Supplemental Figure1

Supplemental Figure2

Supplemental Figure3

Supplemental Figure4

Supplemental table 1

## Data Availability

All gene expression data are available at GSE254614, GSE254615 and GSE254616.
