# Supplementary material for: Metabolic reprogramming regulated by TRAF6 contributes to the leukemia progression
Source: Leukemia. 2024 Apr 12;38(5):1032–45. doi: 10.1038/s41375-024-02245-3 (PMC11073974; doi:10.1038/s41375-024-02245-3)
Supplement: Supplementary file 1 — Supplemental method [file 41375_2024_2245_MOESM1_ESM.docx]

**Supplemental methods**

**Materials**

OGA inhibitor MK-8719 (S8890) was purchased from Selleck. Poly(I:C) (4287) were purchased from Tocris Bioscience. OGT inhibitor OSMI-1 (ab235455) was purchased from Abcam.

**Transfection**

Transfection was performed with HEK293T cells with TransIT-LT1 Transfection Reagent (MIR2305, Mirus) according to the manufactures’ recommendation.

**Lentivirus production and infection**

An inducible knockdown of human TRAF6 was performed in pTRIPZ system (RHS4696-200765571, Horizon Discovery). The leukemic cell lines transduced with pTRIPZ were generated by infection with the supernatants from transfected HEK293T cells in the presence of 4mg/mL of polybrene (TR-1003-G, Millipore). Infected cells were selected in culture with 1ug/mL of puromycin (61-385-RA, Fisher Scientific). Doxycycline (DOX)(BP26535, Fisher Scientific) was used to induce knockdown of TRAF6. For leukemic cell lines expressing shTRAF6 (SHCLNG-NM_004620) or shCtrl (SHC016-EA), pLKO.1-puro vectors were purchased from Sigma-Aldrich. After transduction of pLKO.1-puro vectors, 1 μg of puromycin was used for selection. Knockdown of human OGT was performed in Lentivirus U6-based shRNA knockdown system (VectorBuilder). The lentiviral shRNA expression vectors were used to express shControl (scrambled control, VB010000-0009mxc) and shRNA for human OGT (VB900070-1346wep, 1353zmp and -1455krx1360cjk). After the transduction, 1 μg of puromycin was used for selection. For overexpression of OGT in leukemic cells, lentivirus gene expression system (VectorBuilder) was used. Leukemic cells expressing control vector (stuffer, VB-900120-5944ndj) or human OGT (NM_181672.3, VB211107-1067twd) were selected in culture with 400ug/mL of G418 (16512-36, Nacalai Tesque Inc.)

**Cell cycle analysis**

Cell Cycle Assay Solution Deep Red (C548, DOJINDO) and Click-iT Plus EdU Pacific blue flow cytometry Assay Kit (C10636, Thermo Fisher) was used for human leukemic cells and murine *MLL-AF9* leukemic cells, respectively. The preparation for analyzing TF-1 cells followed a special protocol. Initially, the cells underwent pre-culture in an IL-3-deprived medium for 24 hours. Subsequently, IL-3 was reintroduced into the culture medium, and the cells were further incubated for an additional 24 hours before proceeding with the cell cycle analysis. The analysis was performed according to manufacturer’s instructions. The Flow cytometric analysis was performed on BD FACSCanto II Flow Cytometer (BD biosciences), and data were analyzed by FlowJo software (BD biosciences).

**Glucose uptake assay**

Glucose Uptake Assay Kit-Green (Dojindo, Cat#UP02) or -Red (Dojindo, Cat#UP03) was used for glucose uptake assay. The analysis was performed according to the manufacture’s protocol using human leukemic cells treated with PBS or DOX (1µg/mL) for 3 days, and murine *MLL-AF9* leukemic cells treated with 1μM 4-OHT for 4 days.

**Monitoring changes in intracellular glucose level**

Glucose-Glo Assay (Promega, Cat#J6021) was used for monitoring changes in intracellular glucose levels. The analysis was performed according to the manufacture’s protocol using human leukemic cells treated with PBS or DOX (1µg/mL) for 3 days, and murine *MLL-AF9* leukemic cells treated with 1μM 4-OHT for 4 days.

**Immunoblotting**

Cell extract was prepared by lysing cells in sodium dodecyl sulfate (SDS) sample buffer (1610747, Bio-Rad) followed by incubation with benzonase (70746, Millipore) on ice for 10 minutes. Samples were boiled at 95 °C for 5 minutes and loaded to SDS-polyacrylamide gel electrophoresis (PAGE) using gradient gels (4561084, 4561085, 4561086, Bio-Rad) at 175V for 42minutes and transferred to PVDF membranes (IPVH00010, Merck) at 100V for 1 hour. Immunoblot analysis was performed with the following antibodies: TRAF6 (Cell Signaling, Cat#8028,), OGT (Proteintech, Cat#11576-2-AP,), OGA (Proteintech, Cat#14711-1-AP,), O-GlcNAc, (abcam, Cat#ab2739; Active Motif, Cat#61453) and GAPDH (Cell Signaling, Cat#2118). Primary antibodies were diluted in 5% BSA (A9647, Sigma) or 5% dry fat milk in TBS-T (T9142, TaKaRa) according to manufacturer’s recommendation. Membranes were incubated with primary antibodies overnight at 4℃, and washed 30 minutes in TBS-T before adding secondary diluted to 1:10,000 through 1:5,000 in 5% milk. Imaging was performed Chemi-Doc MP Imaging System (Bio-Rad) or iBright FL1500 Imaging System (Thermo Fisher).

**RNA sequencing**

RNA was isolated using Quick-RNA MiniPrep (Zymo Research, Cat#R1055) or RNeasy Mini Kit (Qiagen, Cat#74106) from human leukemic cells transduced with inducible shTRAF6 treated with PBS or DOX (1µg/mL) for 7 days in biological triplicate. For murine *MLL-AF9* leukemic cells, RNA was extracted with RNeasy Mini Kit (Qiagen, Cat#74106) after the treatment of 1μM of 4-OHT for 4 days in biological triplicate. The RNA samples from HEL, MV4;11 cells were submitted to the DNA Sequencing and Genotyping Core at CCHMC (USA). RNA libraries were prepared according to the Illumina TruSeq Stranded mRNA (polyA capture) library protocol by the DNA Sequencing and Genotyping Core at CCHMC. Libraries were sequenced at an average depth of 30M paired-end 100 nucleotide reads. The samples form TF-1, THP-1, MOLM14, and murine *MLL-AF9* cells were submitted to Kazusa DNA Research Institute (Japan). Total 200 ng of RNA was used for the 3′mRNA library preparation with QuantSeq 3′ mRNA-Seq Library Prep Kit FWD (LEXOGEN, Cat#015.384) according to the manufacture`s protocol. The pooled libraries were loaded on the Illumina Nextseq500 platform and analyzed by 75bp single read. Adaptor sequences were trimmed from the raw RNA-seq reads with fastp (v 0.23.1). Trimmed reads of each sample were mapped to the reference human genome (hg38) or mouse genome (mm10) by using STAR (v 2.3.1) and normalized to 1 million reads in the original library. Genes with an average of 10 or more reads in either group were subjected for further analysis. The analysis of RNA sequencing was performed with iGeak^1^.

**Gene set enrichment analysis**

We performed GSEA as described^2^. Patients with AML were stratified based on low and high TRAF6 expression as defined by: TRAF6 high, Z score > 1; TRAF6 low, Z score < 1.

**References**

1. Choi K, Ratner N. iGEAK: an interactive gene expression analysis kit for seamless workflow using the R/shiny platform. *BMC Genomics* 2019 Mar 6; **20**(1)**:** 177.

2. Subramanian A, Tamayo P, Mootha VK, Mukherjee S, Ebert BL, Gillette MA*, et al.* Gene set enrichment analysis: a knowledge-based approach for interpreting genome-wide expression profiles. *Proc Natl Acad Sci U S A* 2005 Oct 25; **102**(43)**:** 15545-15550.
