# Supplementary material for: Metabolic reprogramming regulated by TRAF6 contributes to the leukemia progression
Source: Leukemia. 2024 Apr 12;38(5):1032–45. doi: 10.1038/s41375-024-02245-3 (PMC11073974; doi:10.1038/s41375-024-02245-3)
Supplement: Supplementary file 2 — Supplemental Figure1 [file 41375_2024_2245_MOESM2_ESM.pdf]

Supplemental Figure 1

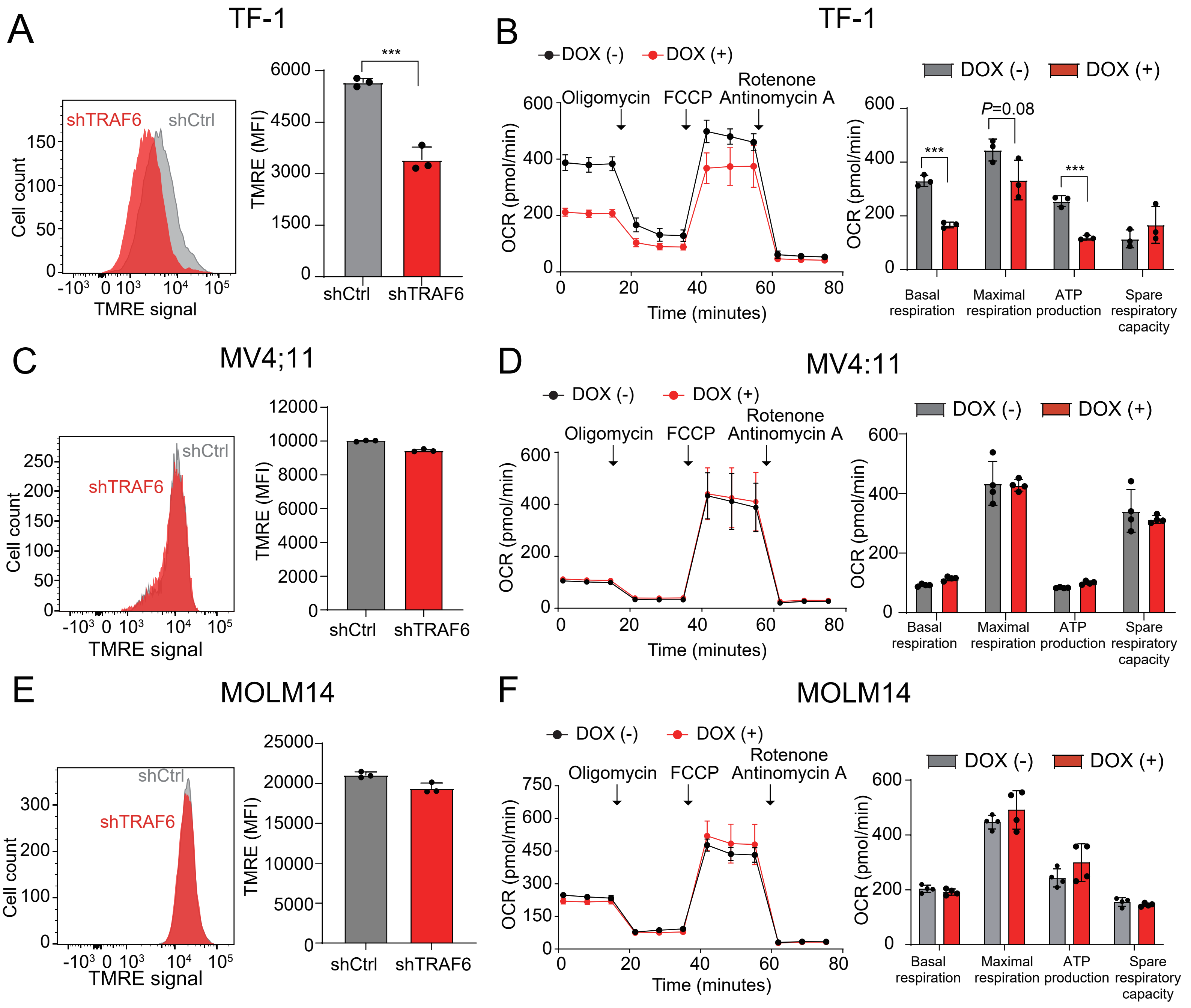

Supplemental Fgure 1. Evaluation of TRAF6 loss in the changes in the mitochindrial function of human leukemia cells. **(A, C and E)** Representative flow cytometry histograms of mitochondrial TMRE levels in TF-1 (A), MV4;11 (C) and MOLM14 (E) cells expressing shTRAF6 or shControl (shCtrl) (left panel). Median fluorescent intensity (MFI) of TMRE observed from TF-1 (A), MV4;11 (C) and MOLM14 (E) cells expressing shTRAF6 or shCtrl (right panel). Data are presented as the means ± SD from biological triplicates. Results are representative of two independent assays. **(B, D and F)** Oxygen consumption rate (OCR) in TF-1 (B), MV4;11 (D) and MOLM14 (F) cells transduced with the inducible shTRAF6. Cells were sequentially treated with oligomycin, fluoro-carbonyl cyanide phenylhydrazone (FCCP), and rotenone/antimycin A at the indicted time points (left panel). Basal respiration, maximal respiration, ATP production and spare respiratory capacities of HEL cells transduced with the inducible shTRAF6 calculated from the data of the right panels. Data are presented as the means ± SD from technical replicates (n = 4). Results are representative of two independent assays. ***, *P* < 0.001.
