# Supplementary material for: Metabolic reprogramming regulated by TRAF6 contributes to the leukemia progression
Source: Leukemia. 2024 Apr 12;38(5):1032–45. doi: 10.1038/s41375-024-02245-3 (PMC11073974; doi:10.1038/s41375-024-02245-3)
Supplement: Supplementary file 3 — Supplemental Figure2 [file 41375_2024_2245_MOESM3_ESM.pdf]

Supplemental Figure 2

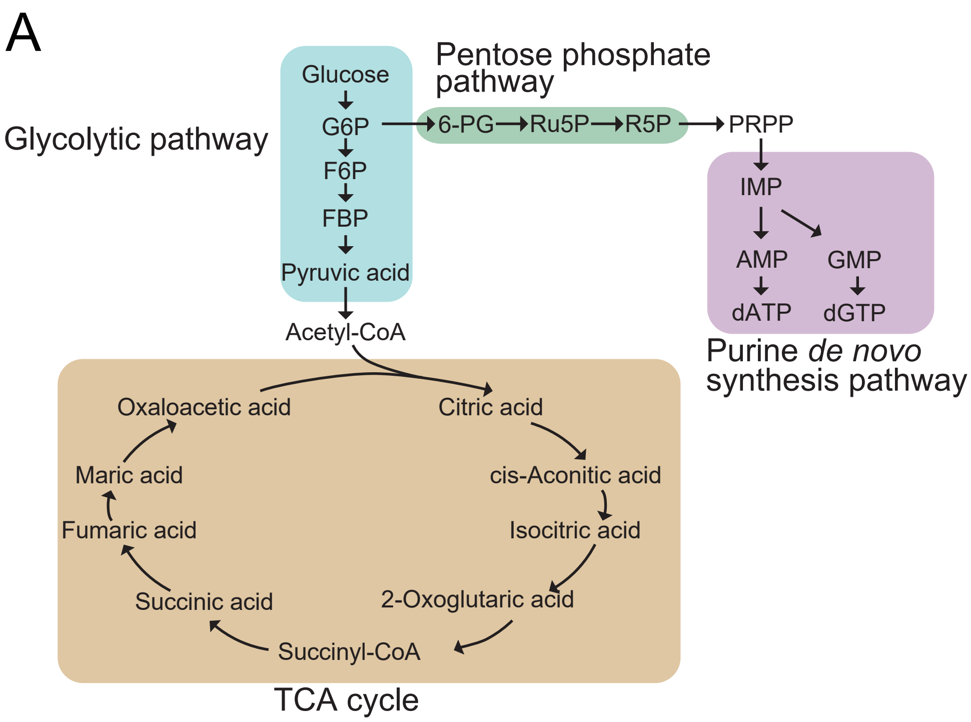

Supplemental Figure 2. Metabolic map of glycolysis, TCA cycle, pentose phosphate pathway and purine de novo synthesis pathway. **(A)** G6P, glucose 6-phosphate; F6P, fructose 6-phosphate; FBP, fructose 1,6-diphosphate; 6-PG, 6-phosphogluconic acid; Ru5P, ribulose 5-phosphate; R5P, ribose 5-phosphate; PRPP, phosphoribosyl pyrophosphate; IMP, inosine monophosphate; dATP, deoxyadenosine triphosphate; dGTP, deoxyguanosine triphosphate.
