# Supplementary material for: Metabolic reprogramming regulated by TRAF6 contributes to the leukemia progression
Source: Leukemia. 2024 Apr 12;38(5):1032–45. doi: 10.1038/s41375-024-02245-3 (PMC11073974; doi:10.1038/s41375-024-02245-3)
Supplement: Supplementary file 4 — Supplemental Figure3 [file 41375_2024_2245_MOESM4_ESM.pdf]

Supplemental Figure 3

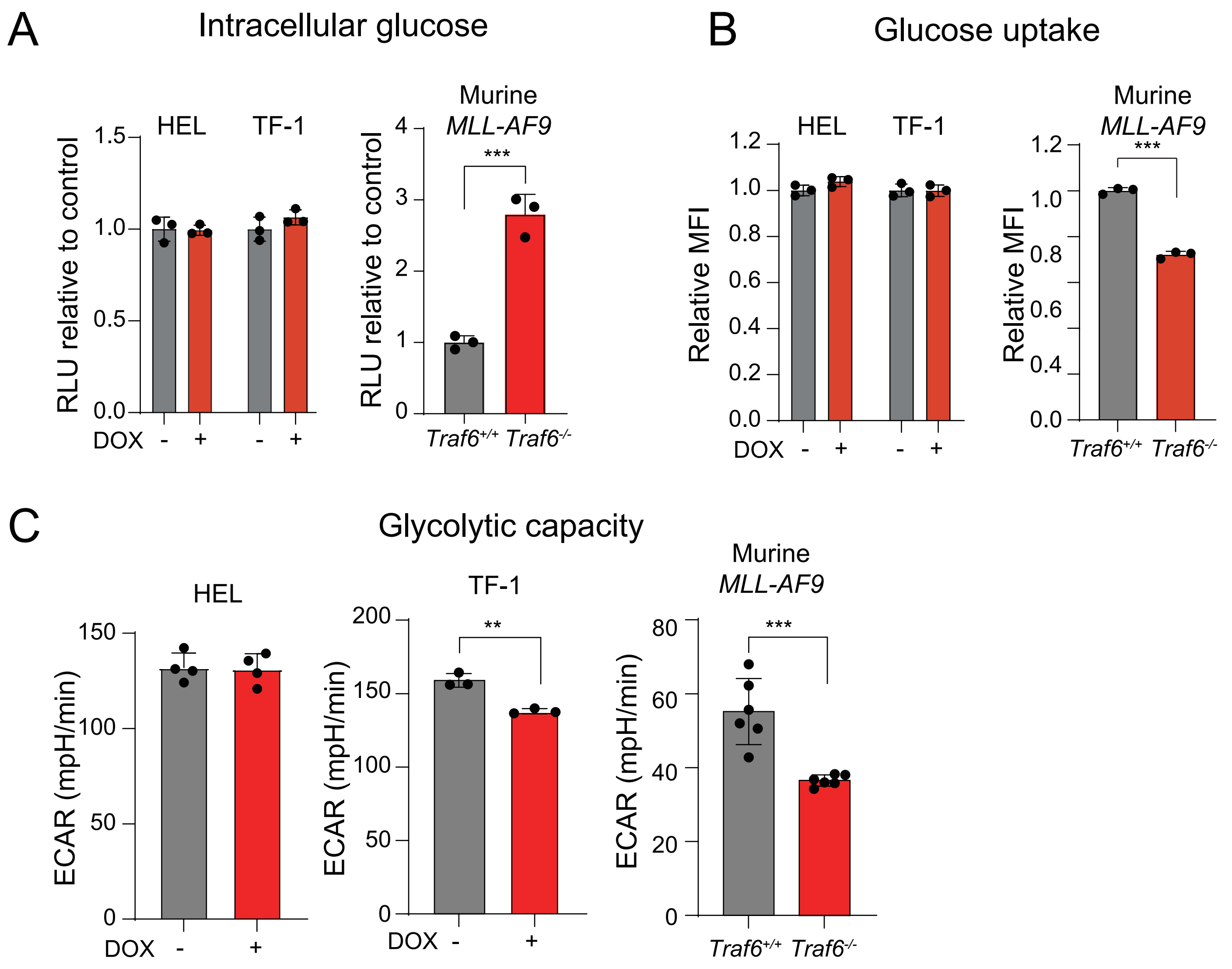

Supplemental Figure 3. Evaluation of TRAF6 loss on mitochondrial function changes in human leukemia cells. **(A)** Intracellular glucose levels in HEL and TF-1 transduced with inducible shTRAF6, treated with or without DOX (1µg/mL) for 3days (left panel), and in *MLL-AF9;Traf6*$^{+/+}$ and *MLL-AF9;Traf6*$^{-/-}$ leukemic cells (right panel). Data are presented as the means ± SD from biological replicates (n = 3). Results are representative of two independent assays.**(B)** Glucose uptake capacity of HEL and TF-1 transduced with inducible shTRAF6, treated with or without DOX (1µg/mL) for 3days (left panel), and of *MLL-AF9;Traf6*$^{+/+}$ and *MLL-AF9;Traf6*$^{-/-}$ leukemic cells (right panel). Data are presented as the means ± SD from biological replicates (n = 3). Results are representative of two independent assays. **(C)** Glycolytic capacity of HEL (left panel) and TF-1 (middle panel) cells transduced with inducible shTRAF6, treated with or without DOX (1µg/mL) for 3days, and of *MLL-AF9;Traf6*$^{+/+}$ and *MLL-AF9;Traf6*$^{-/-}$ leukemic cells (right panel). Data are presented as the means ± SD from technical replicates (n = 3-6). Results are representative of two independent assays. **, $P < 0.01$; ***, $P < 0.001$.
