# Supplementary material for: Metabolic reprogramming regulated by TRAF6 contributes to the leukemia progression
Source: Leukemia. 2024 Apr 12;38(5):1032–45. doi: 10.1038/s41375-024-02245-3 (PMC11073974; doi:10.1038/s41375-024-02245-3)
Supplement: Supplementary file 5 — Supplemental Figure4 [file 41375_2024_2245_MOESM5_ESM.pdf]

Supplemental Figure 4

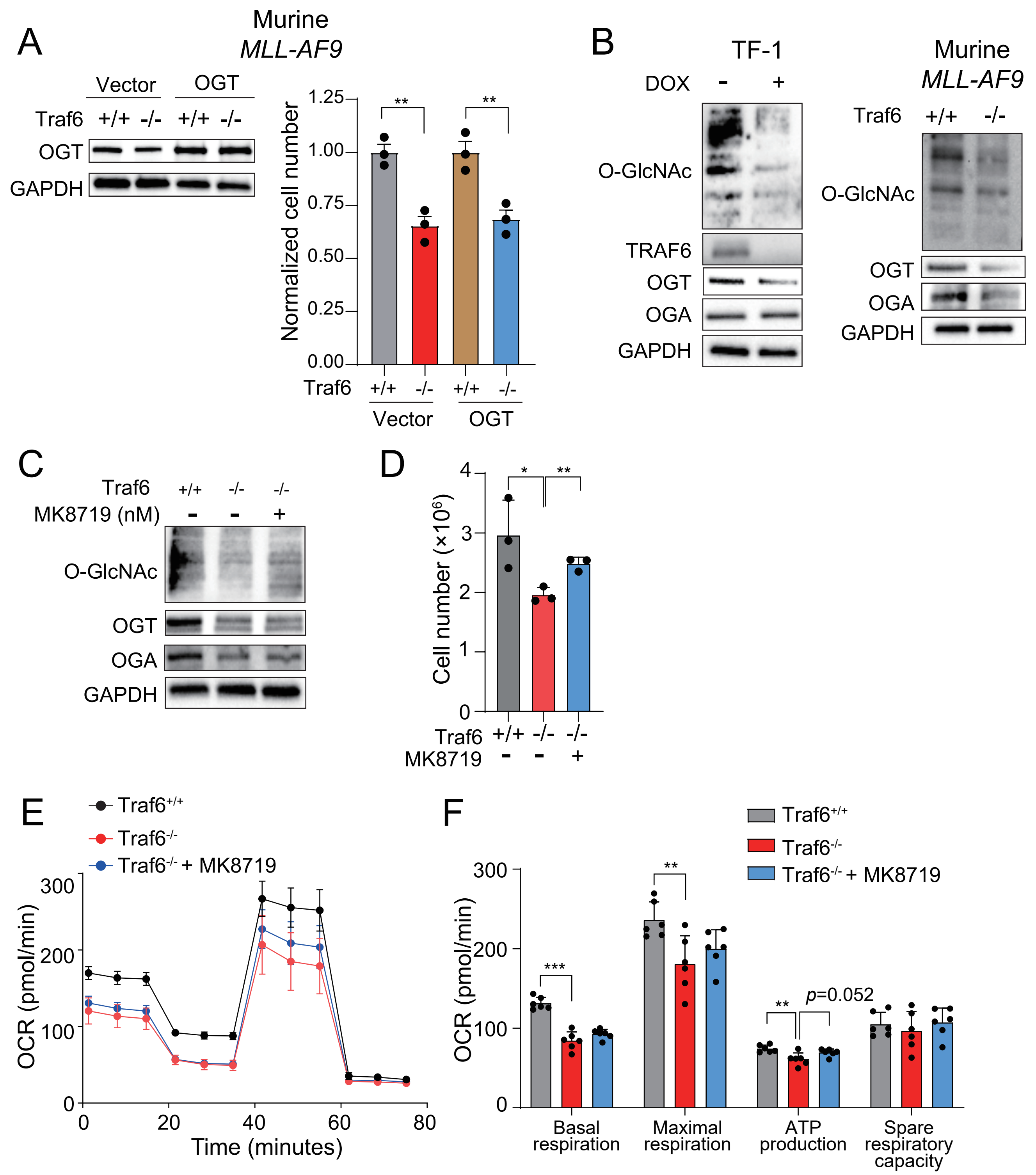

Supplemental Figure 4. Fine regulation of O-GlcNac modification in leukemia by TRAF6. (A) Immunoblot analysis of OGT in *MLL-AF9;Traf6*$^{+/+}$ and *MLL-AF9;Traf6*$^{-/-}$ leukemic cells expressing either control vector or cDNA of OGT (left panel). Viable cell growth of the cells was assayed by trypan blue exclusion(right panel). The cell number relative to *Traf6*$^{+/+}$ cells was evaluated 72 hours after equal number of the cells were seeded. Data are presented as the means ± SD from technical triplicates. Results are representative of two independent assays. **(B)** Immunoblot analysis of TF-1 cells transduced with the inducible shTRAF6, treated with or without DOX (1μg/mL) for 3 days (left panel), and of *MLL-AF9;Traf6*$^{+/+}$ and *MLL-AF9;Traf6*$^{-/-}$ leukemic cells (right panel). **(C)** Immunoblotting of *MLL-AF9;Traf6*$^{+/+}$ and *MLL-AF9;Traf6*$^{-/-}$ leukemic cells, treated with or without 800nM of MK8719.**(D)** Cell viability was evaluated in *MLL-AF9;Traf6*$^{+/+}$ and *MLL-AF9;Traf6*$^{-/-}$ leukemic cells, cultured with or without MK8719 for 4 days. **(E)** OCR in *MLL-AF9;Traf6*$^{+/+}$ and *MLL-AF9;Traf6*$^{-/-}$ leukemic cells treated with or without 800nM of MK8719. Cells were sequentially treated with oligomycin. FCCP, and rotenone/antimycin A at the indicted time points. Data are presented as the means ± SD from technical replicate analyses (n = 6). Results are representative of two independent assays. **(F)** Basal respiration, maximal respiration, ATP production and spare respiratory capacities of *MLL-AF9;Traf6*$^{+/+}$ and *MLL-AF9;Traf6*$^{-/-}$ leukemic cells treated with or without 800nM of MK8719, calculated from the data of (E). Data are shown as the means ± SD (n = 6). *, P < 0.05; **, < 0.01; ***, P < 0.001.
